# A Novel Silent Mutation in the *L1CAM* Gene Causing Fetal Hydrocephalus Detected by Whole-Exome Sequencing

**DOI:** 10.3389/fgene.2019.00817

**Published:** 2019-09-11

**Authors:** Yixi Sun, Yanfeng Li, Min Chen, Yuqin Luo, Yeqing Qian, Yanmei Yang, Hong Lu, Fenlan Lou, Minyue Dong

**Affiliations:** ^1^Department of Reproductive Genetics, Women’s Hospital, School of Medicine, Zhejiang University, Hangzhou, China; ^2^Key Laboratory of Reproductive Genetics, Ministry of Education (Zhejiang University), Hangzhou, China; ^3^Key Laboratory of Women’s Reproductive Health of Zhejiang Province, Hangzhou, China; ^4^Department of Ultrasound, Women’s Hospital, School of Medicine, Zhejiang University, Hangzhou, China; ^5^Department of Diagnostic Radiology, Women’s Hospital, School of Medicine, Zhejiang University, Hangzhou, China

**Keywords:** hydrocephalus, *L1CAM*, whole-exome sequencing, silent mutation, splicing mutation

## Abstract

X-linked hydrocephalus (XLH), a genetic disorder, has an incidence of 1/30,000 male births. The great proportion of XLH is ascribed to loss-of-function mutations of L1 cell adhesion molecule gene (*L1CAM*), but silent mutations in *L1CAM* with pathogenic potential were rare and were usually ignored especially in whole-exome sequencing (WES) detection. In the present study, we describe a novel silent *L1CAM* mutation in a Chinese pregnant woman reporting continuous five times pregnancies with fetal hydrocephalus. After fetal blood sampling, we found c.453G > T (p.Gly151 = ) in the *L1CAM* gene of the fetus by WES; RT-PCR of the messenger RNA (mRNA) from cord blood mononuclear cells and subsequent sequence analysis identified the mutation created a potential 5′ splice site consensus sequence, which would result in an in-frame deletion of 72 bp from exon 5 and 24 amino acids of the *L1CAM* protein. Heterozygous mutations were confirmed in analyzing DNA and mRNA from peripheral blood mononuclear cells of the woman, and a severe L1 syndrome was confirmed by fetal ultrasound scan and MRI. Our study first indicated c.453G > T (p.Gly151 = ) in *L1CAM* could be disease causing for hydrocephalus, which would aid in genetic counseling for the prenatal diagnosis of hydrocephalus. Meanwhile, it suggested some silent mutations detected in WES should not be ignored; splicing predictions of these mutations were necessary.

## Introduction

The L1 cell adhesion molecule gene (*L1CAM*) is a neuronal cell adhesion molecule belonging to the immunoglobulin superfamily; it possesses key functions in the development of the nervous system (Itoh and Fushiki, 2015). Mutations in *L1CAM* have been related to X-linked neurological syndromes, which are summarized as L1 diseases. They are classified as follows: X-linked hydrocephalus (XLH) due to stenosis of the aqueduct of Sylvius (HSAS), MASA syndrome (intellectual disability, aphasia, shuffling gait, adducted thumbs), spastic paraparesis type 1 (SP1), and X-linked agenesis of corpus callosum (ACC) (Weller and Gartner, 2001; Itoh and Fushiki, 2015).

About 282 disease-causing mutations (DMs) in the *L1CAM* gene have been reported in HGMD^®^ Professional 2019.2 (https://portal.biobase-international.com/hgmd/pro/all.php). Alterations in the *L1CAM* gene are varied; mutation data analyses from 282 patients disclose 51% missense and nonsense mutations, 25% deletions, 5% insertions, and 19% splice site changes, but silent mutations in *L1CAM* with pathogenic potential were rare, and silent mutations were often ignored especially in whole-exome sequencing (WES) detection.

In this study, using WES, we screened the fetal DNA of a Chinese pregnant woman who has reported five continuous pregnancies with fetal hydrocephalus; we only found a novel silent mutation c.453G > T (p.Gly151 = ) in the *L1CAM* gene. Interestingly, through further analysis, we indicated the silent mutation created a potential 5′ splice site consensus sequence, which would result in an in-frame deletion of 72 bp from exon 5 and 24 amino acids of the *L1CAM* protein.

## Case Presentation

A 28-year-old healthy woman was referred to our clinic after four voluntary terminations of pregnancy due to fetal hydrocephalus at other hospitals. All fetuses were male. When arriving at our hospital (Women’s Hospital, School of Medicine, Zhejiang University, Zhejiang, China), she was already on her fifth pregnancy at 24 weeks of gestation, with a fetal hydrocephalus by image examinations. To explore the genetic cause, fetal blood sampling was conducted at 26 weeks of gestational age. Conventional cytogenetic studies were performed for both fetal and parental samples, and the fetal sample was further analyzed by single-nucleotide polymorphism (SNP) array and WES.

This study was carried out in accordance with the recommendations of the Ethics Committee of Women’s Hospital, School of Medicine Zhejiang University, and informed consent was acquired from all the participants of this study in accordance with the Declaration of Helsinki. The study protocol was approved by the Review Board of the Women’s Hospital, School of Medicine, Zhejiang University in China.

## Materials and Methods

### Karyotype and SNP Array

The karyotypes of fetal cord blood and peripheral cord blood were determined by conventional karyotyping of at least 30 blood lymphocytes, which were arrested at metaphase by colchicines. G-banding karyotypes of cultured cells were performed at the 320–400-band level with a resolution of around 10 Mb. SNP array was performed by the CytoScan^™^ HD array (Affymetrix, USA) according to the manufacturer’s instruction, with around 2,600,000 markers including 750,000 SNP probes and 1,900,000 non-polymorphism probes for comprehensive whole-genome coverage. Data were analyzed by the Chromosome Analysis Suite (ChAS) software (Affymetrix, Santa Clara, CA) based on the GRCh37/hg19 assembly. The reporting threshold of the copy number result was set at 500 kb with a marker count of ≥50 for gains and at 200 kb with a marker count of ≥50 for losses.

### Whole-Exome Sequencing

The main part of WES was provided by the Beijing Genomics Institute. Genomic DNA was extracted by a DNeasy Blood Kit (Qiagen, CA) and then was fragmented by Covaris LE220 (Massachusetts, USA) to generate a paired-end library (200–250 bp). All amplified libraries were performed on the BGISEQ-500 platform, the single-strand DNA was mixed with MGIEasy^™^ DNA Library Prep Kit V1 (BGI, Shenzhen, China) and then sequenced using 100SR chemistry with BGISEQ-500RS high-throughput sequencing Kit (BGI, Shenzhen, China).

Clean reads (with a length of 90 bp) derived from targeted sequencing and filtering were then aligned to the human genome reference (hg19) using the Burrows-Wheeler Aligner (BWA) Multi-Vision software package (Li and Durbin, 2009). After alignment, the output files were used to perform sequencing coverage and depth analysis of the target region, single-nucleotide variants (SNVs), and indel calling, we used the GATK software to detect SNVs and indels (McKenna et al., 2010), all SNVs and indels were filtered and estimated *via* multiple databases, including the National Center for Biotechnology Information (NCBI) Single-Nucleotide Polymorphism Database (dbSNP), HapMap, 1000 Genomes Project dataset, and database of 100 Chinese healthy adults. We used Condel, SIFT, PolyPhen-2, LRT, Mutation Taster, and PhyloP to predict the effect of variants. Pathogenic variants are assessed under the protocol issued by the American College of Medical Genetics and Genomics (ACMG) (Richards et al., 2015). The Human Gene Mutation Database (HGMD) was used to screen mutations. All potential pathogenic variants were validated using Sanger sequencing methods.

### RNA Extraction, PCR, and Sequencing

Peripheral blood mononuclear cells (PMBCs) and cord blood mononuclear cells (CBMCs) were isolated by Ficoll density gradient separation. Total RNA was extracted from PMBCs and the CBMCs using TRIzol (Takara, Japan). Extracted total RNAs were reverse-transcribed using RT Kit (Takara, Japan). PCR was performed using GoldStar Best Master Mix (CWBIO, Beijing). Primer sequences are listed: L1CAM-DNA-5F, CCCACCCGTCCTTTCCTA; L1CAM-DNA-5R, CGCTCGTCCTGCTTGATGT; L1CAM-mRNA-4-6-F, GGTGTCCACTTCAAACCCAA; and L1CAM-mRNA-4-6-R, GCGGCTTCCTGTCAATCA. Sanger sequencing was performed by an ABI 3130 DNA analyzer.

## Results

**Figure 1:**
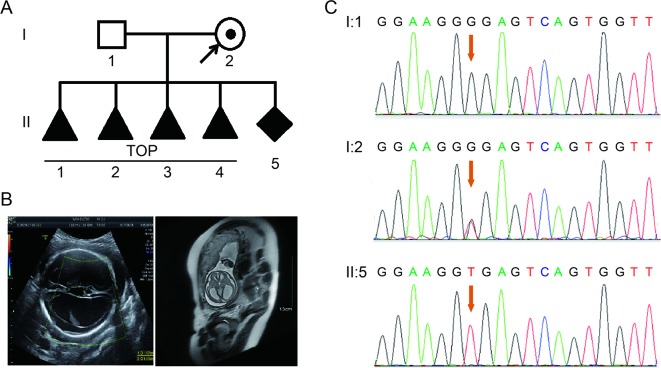
**(A)** Pedigree of the family. TOP, termination of pregnancy. **(B)** Imaging examinations of the fetus. Fetal ultrasound scan and fetal MRI showed there was severe hydrocephalus in the fetus. **(C)** Sequence analysis of genomic DNA from family members. The genotypes of *L1CAM* were wild type, c.453G > T Het, and c.453G > T Hom, in I:1 (husband), I:2 (pregnant woman), and II:5 (fetus). The mutation is indicated by the red arrows.

A 28-year-old healthy woman was referred to our clinic after four voluntary terminations of pregnancy due to fetal hydrocephalus. All fetuses were male (**Figure 1A**). The familial pedigree seemed to show XLH. She was already on here fifth pregnancy at 26 weeks of gestation. Fetal ventriculomegaly was detected by fetal ultrasound scan and MRI, which consistently demonstrated the presence of hydrocephalus. They showed that the bilateral cerebral ventricle and the third ventricle were obviously dilated, and there was severe hydrocephalus in the intracerebral and agenesis of the corpus callosum (**Figure 1B**).

In order to explore the possible genetic cause, we performed karyotype analysis and SNP array to analyze the fetal blood sampling and found no positive findings. Choroidal neovascularization (CNV) result has been deposited in the Gene Expression Omnibus (GEO); the accession number is GSE133063, as appended below (https://www.ncbi.nlm.nih.gov/geo/query/acc.cgi?acc=GSE133063).

Then, we detected the fetus by WES. The analytic strategy for finding likely pathogenic variant identification was shown in **Figure S1**. A list of variants (**Table S1**) were obtained through the screening of variant frequencies, mutation status, and inheritance mode. Taking the hydrocephalus-associated genes (HP:0000238, http://compbio.charite.de/hpoweb/showterm?id=HP:0000238#id=HP_0000238) (**Table S2**) into consideration, there was no additional notable mutation except for the silent mutation of c.453G > T in exon 5 of the *L1CAM* gene (NM_000425.3). c.453G > T was not reported in HGMD and ClinVar and was not found in dbSNP, gnomAD, and other datasets. According to the standards and guidelines of the ACMG (Richards et al., 2015), it had not yet reached the criterion of “pathogenic” or “likely pathogenic,” but there was no other potential mutations; we had no choice but to make a further analysis of the silent mutation found.

According to traditional thinking, this base substitution occurred in the third base in codon 151, which encodes a glycine, thereby creating a neutral mutation (p.Gly151 = ). This variant was confirmed in DNA extracted from fetal cord blood and peripheral blood in the couple by Sanger sequencing (**Figure 1C**). The woman carried the heterozygous mutation, and her husband was a wild-type genotype.

**Figure 2:**
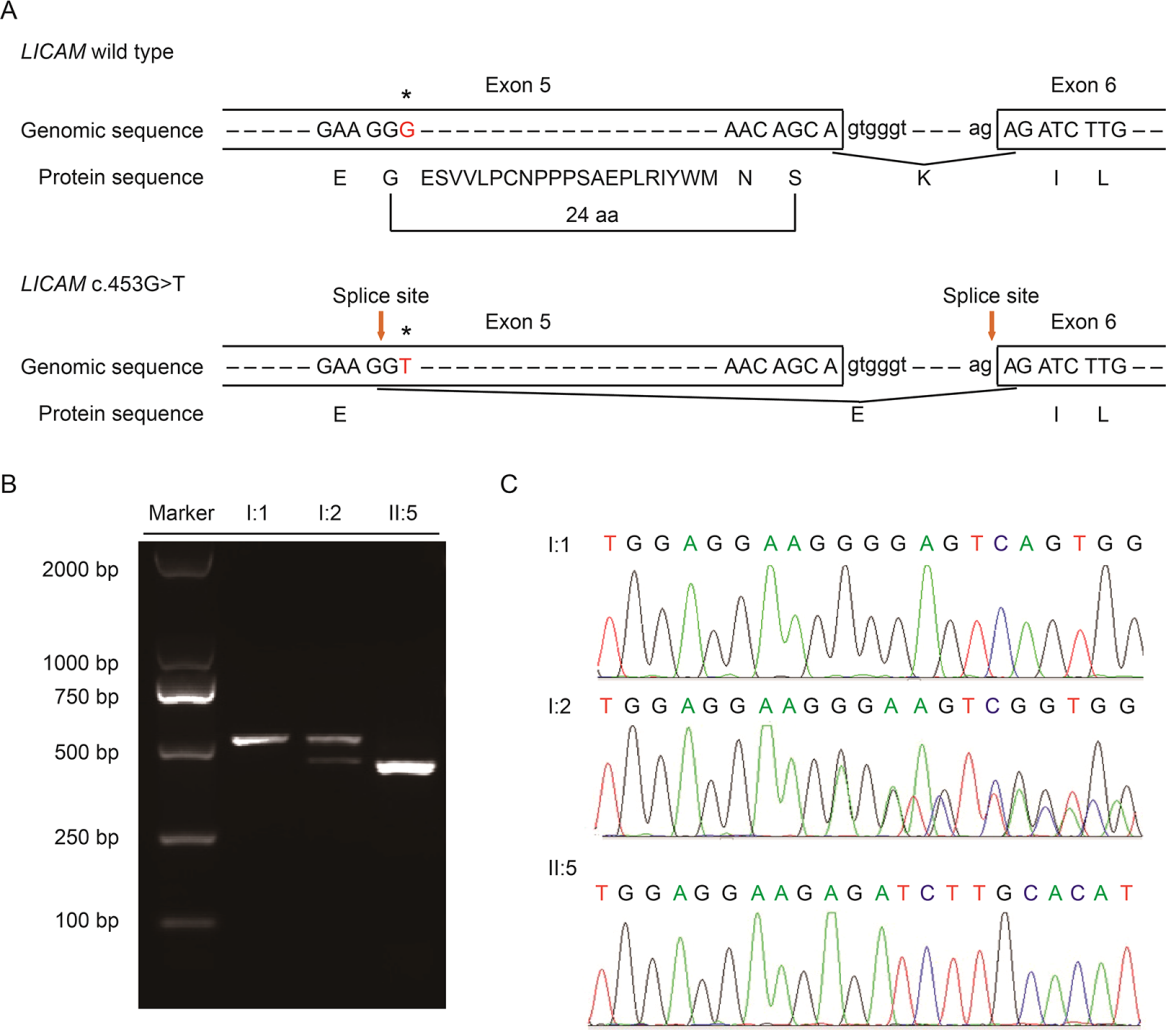
**(A)** Schematic representation of exon 5, intron 6, and exon 6 organization in *L1CAM*. **(B)** RT-PCR analysis of exons 5 and 6 of the *L1CAM* cDNA from peripheral blood mononuclear cells (PMBCs) and cord blood mononuclear cells (CBMCs). Agarose gel electrophoresis of RT-PCR products generated from I:1 (husband), I:2 (pregnant woman), and II:5 (fetus). **(C)** Sequence analysis of the RT-PCR product from PMBCs of the couple and CBMCs of the fetus.

With Mutation Taster (http://www.mutationtaster.org/), c.453G > T was scored as “disease causing.” It showed that protein features might be affected and the splice site might be changed; we were curious about the potential splicing effects of the *L1CAM* function in this silent mutation. The silent mutation was tested using the following online software products: NetGene2 (http://www.cbs.dtu.dk/services/NetGene2/) and NNSplice (http://www.fruitfly.org/seq_tools/splice.html); the 5′ potential splice site was also predicted to be created in the *L1CAM* c.453G > T mutation using the software products (**Figure S2**). The results showed that this silent mutation created a potential 5′ splice site 72 bp upstream from the normal exon 6/intron 6 splice site (**Figure 2A**). If this is the case, we can find the length change of *L1CAM* messenger RNA (mRNA) between I:2 and II:5 (**Figure 2A**). RT-PCR was performed using primers designed to amplify exons 4–6 in *L1CAM* mRNA. Indeed, the results showed a short band of truncation in fetal cDNA PCR (II:5), while the band amplified from *L1CAM* mRNA contained the expected long band in husband cDNA PCR (I:1) and long/short bands in the pregnant woman cDNA PCR (I:2) (**Figure 2B**). Direct sequencing of the amplified fragment showed that the deletion involved the last 72 bp of exon 5 in male fetal cDNA (the woman was a carrier) (**Figure 2C**). We got the crucial pathogenic evidence.

**Figure 3:**
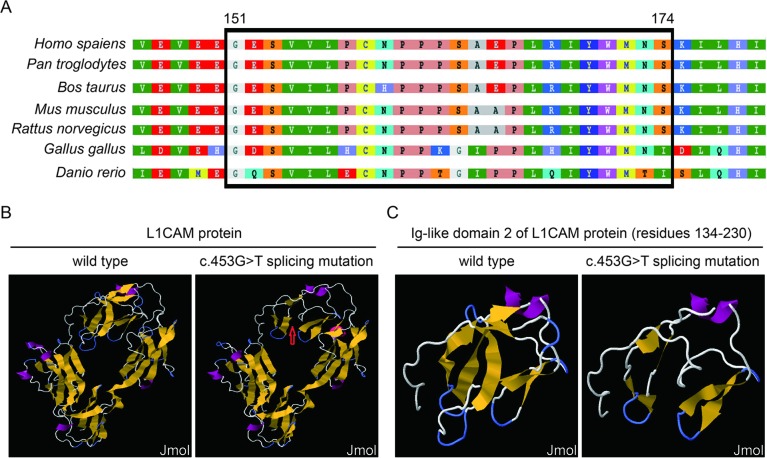
**(A)** Alignment of multiple L1CAM protein sequences across species. The *L1CAM* c.453G > T resulted in 24 amino acids of L1CAM protein (residues 151–174) missing in the conserved amino acid region in different species. The black column shows the missing amino acids. **(B)** The structures of wild-type and c.453G > T splicing mutation L1CAM protein as predicted by the software CPHmodels-3.2 Server. **(C)** The structures of immunoglobulin-like (Ig-like) domain 2 (residues 134–230) of wild-type and splicing mutation L1CAM protein.

This silent mutation resulted in 24 amino acids of L1CAM protein (residues 151–174); Lys (K) was substituted by Glu (E) at codon 175 (**Figure 2A**). There were alignment of multiple L1CAM protein sequences across several species and conservation of the missing amino acids in L1CAM across mammals: *Homo sapiens*, *Pan troglodytes*, *Bos taurus*, *Mus musculus*, and *Rattus norvegicus* (**Figure 3A**). Wild-type and c.453G > T splicing mutation L1CAM proteins were predicted by the software CPHmodels-3.2 Server (http://www.cbs.dtu.dk/services/CPHmodels/) (**Figure 3B**). Immunoglobulin-like (Ig-like) domain 2 (residues 134–230) of wild-type and splicing mutation L1CAM proteins is shown in **Figure 3C**. *L1CAM* c.453G > T splicing mutation altered the protein structure, especially the Ig-like domain 2

## Discussion

Silent mutations were often detected by WES, but insufficient attention has been paid, leading to the omission of DMs. In this study, we employed WES to explore the genetic cause of a Chinese family with hydrocephalus but only found a novel silent mutation in *L1CAM*, which forced us to make a further analysis. Fortunately, we proved that the silent mutation created a new 5’ splice site and was a DM.

Mutations in *L1CAM* can cause an X-linked L1 disease, but clinical symptoms are variable; mutations produce unexpected phenotypes. In the study, the five suffering fetuses are all males, which is consistent with an inheritance pattern. The fetal ultrasound scan and MRI show a typical L1 disease, including XLH and agenesis of the corpus callosum. It improves our understanding on the genotype–phenotype correlation of L1CAM.

*L1CAM* c.453G > T (p.Gly151 = ) was initially thought to have no effect on the protein sequence. But other silent mutations, c.924C > T (p.Gly308 = ) and c.645C > T (p.Gly215 = ), in the *L1CAM* gene have been reported to be DMs (Du et al., 1998; Vos et al., 2010). The c.924C > T mutation resulted in the activation of a new splice site 69 bp 5′ to the normal exon 8/intron 8 donor splice site, and it has been declared as a “disease-causing” site for hydrocephalus (Du et al., 1998). For c.645C > T in *L1CAM*, 51 bp was deleted with the activation of a new exon 6/intron 6 donor splice (Vos et al., 2010). Our present study was similar; the mutation of c.453G > T created a potential 5′ splice site upstream from the normal exon 5/intron 5 splice site. All these silent mutations created new donor splice sites, resulting in the exon being skipped. It reminded us to pay close attention to these silent mutations, which may affect splicing of proteins.

As a transmembrane glycoprotein and a member of the immunoglobulin superfamily of cell adhesion molecules, the L1CAM protein can interact at the cell surface with a number of different glycoproteins, and homophilic binding is probably its principal mode of interaction (Wei and Ryu, 2012). The studies on the crystal structure of Ig-like domains 1–4 in neurofascin suggested that many pathological L1 mutations affect conserved amino acid residues within these domains and interfere with homophilic interactions (Liu et al., 2011), especially as verified by the function research of Ig-like domain 2 (Zhao et al., 1998). In our study, we speculated that *L1CAM* c.453G > T altered Ig-like domain 2 in the extracellular part of the L1CAM protein, leading to the abnormal extracellular interaction, failing to start initiating downstream the signaling pathway. A further study dedicated to the mass spectrometry of this L1CAM variant would clarify specifically what molecular ensemble is produced in the cell.

In summary, through WES, we reported a novel silent mutation c.453G > T in *L1CAM* which produces a 5′ splice site responsible for hydrocephalus. This abnormal protein variant was predicted to alter Ig-like domain 2, which might affect L1CAM protein homophilic binding. In addition, we performed prenatal genetic diagnosis for the pregnant woman reporting five continuous pregnancies with hydrocephalus. Meanwhile, it suggested some silent mutations detected in WES should not be ignored; splicing predictions of these mutations were necessary. It provided a new genetic basis for prenatal diagnosis and pre-implantation prenatal diagnosis of hydrocephalus.

## Data Availability

Publicly available datasets were analyzed in this study. This data can be found here: GSE133063(https://www.ncbi.nlm.nih.gov/geo/query/acc.cgi?acc=GSE133063).

## Ethics Statement

The studies involving human participants were reviewed and approved by the Review Board of the Women’s Hospital, School of Medicine, Zhejiang University in China. Written informed consent to participate in this study was provided by the participants’ legal guardian/next of kin. Written informed consent was obtained from the individual(s), and minor(s)’ legal guardian/next of kin, for the publication of any potentially identifiable images or data included in this article.

## Author Contributions

YS, YLi, MC, YLu, YQ, and YY conducted experiments. YS prepared the figures. MC and YLi analyzed the WES data. YLu and YQ performed karyotype analysis and SNP array. YY recruited samples. HL and FL provided imaging examinations. YS and MD wrote the manuscript. All authors read and approved the final manuscript.

## Funding

This study was supported by the National Natural Science Foundation of China (Grant No. 81801441), the Key Research and Development Program of the Zhejiang province (Grant No. 2019C03025), the National Key Research and Development Program of China (Grant No. 2016YFC1000703), and the Medical Scientific Research Foundation of Zhejiang Province (Grant No. 2014KYA246).

## Conflict of Interest Statement

The authors declare that the research was conducted in the absence of any commercial or financial relationships that could be construed as a potential conflict of interest.

## Supplementary Material

Figure S1 Analytic strategy for finding likely pathogenic variant identification by WES.

Figure S2 Donor splice sites predicted by NetGene2 and NNSplice.

Table S1 List of variants through the screening of variants frequencies, mutation status, and inheritance mode.

Table S2 List of hydrocephalus-associated genes (Export for HP:0000238).
