# Supplementary figures and images for: A Novel Silent Mutation in the L1CAM Gene Causing Fetal Hydrocephalus Detected by Whole-Exome Sequencing
Source: Front Genet. 2019 Sep 11;10:817. doi: 10.3389/fgene.2019.00817 (PMC6749797; doi:10.3389/fgene.2019.00817)

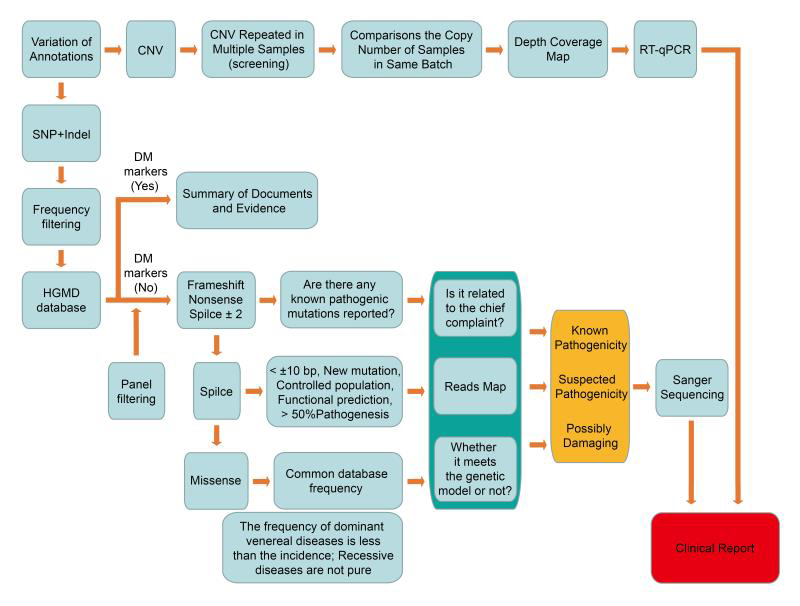

Supplement: Figure S1 — Analytic strategy for finding likely pathogenic variant identification by WES. [file Image_1.tif]

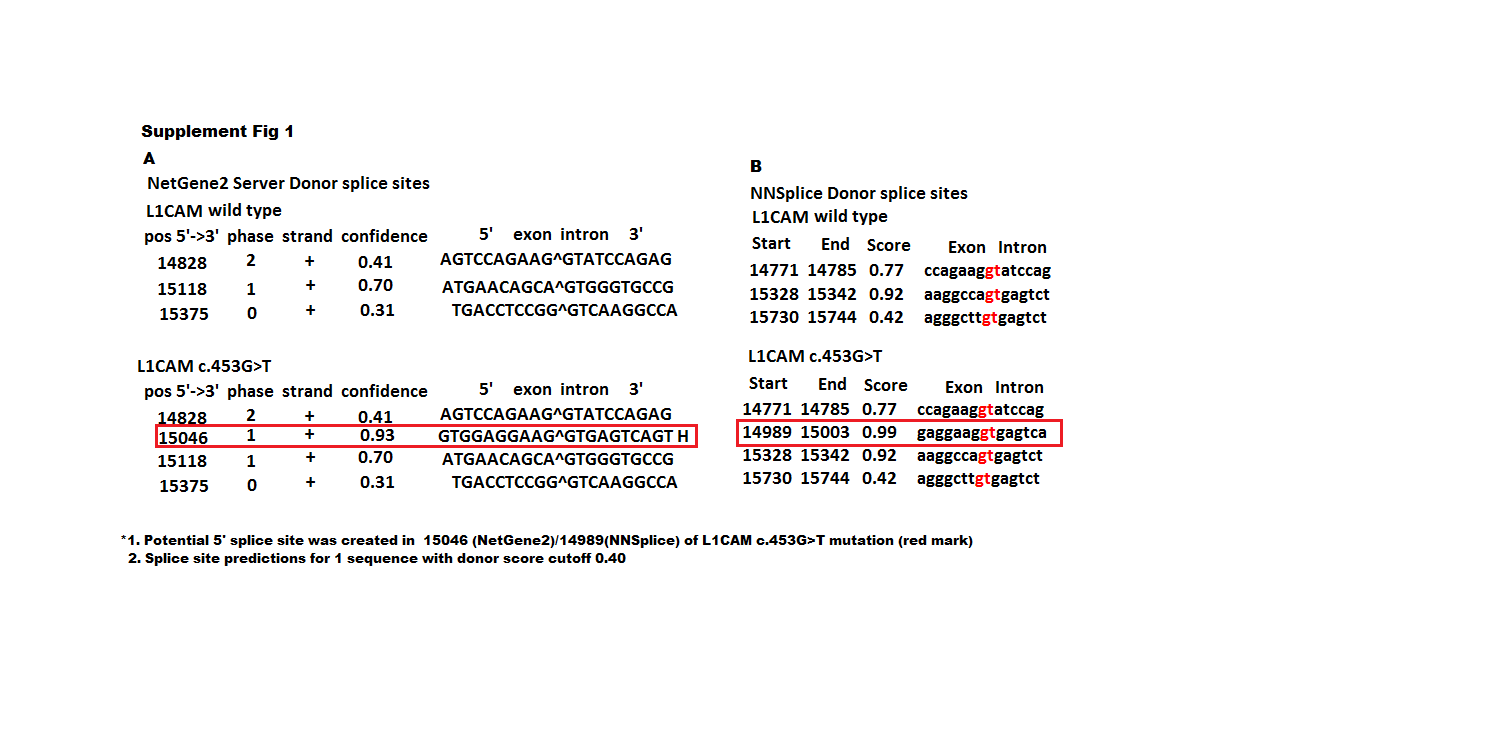

Supplement: Figure S2 — Donor splice sites predicted by NetGene2 and NNSplice. [file Image_2.tif]
